# Spatiotemporal Crosstalk Between Oocyte and the Microenvironment Governs Preovulatory Follicle Aging

**DOI:** 10.1111/acel.70302

**Published:** 2025-11-23

**Authors:** Xin Yi Koh, Kah Junn Tan, Zeng Hao Lim, Shi Chee Ong, Sophie Emma Tan, Jing Xuan Lu, Zhongwei Huang, Jun Wei Pek

**Affiliations:** ^1^ Temasek Life Sciences Laboratory Singapore Singapore; ^2^ Department of Biological Sciences National University of Singapore Singapore Singapore; ^3^ National University of Singapore Singapore Singapore; ^4^ Singapore Polytechnic Singapore Singapore; ^5^ Department of Obstetrics & Gynaecology National University Hospital Singapore Singapore; ^6^ NUS Bia‐Echo Asia Centre for Reproductive Longevity and Equality (ACRLE), Yong Loo Lin School of Medicine National University of Singapore Singapore Singapore

**Keywords:** aging, circRNA, *drosophila*, granulosa cell, mitochondria, PIGBOS, Preovulatory oocyte, *sestrin*

## Abstract

Preovulatory follicle aging is the period between formation and ovulation of a mature follicle. Previous studies had shown that mammalian preovulatory follicle aging is associated with chromosomal abnormalities and developmental defects such as decreased implantation, increased malformation and mortality and lower embryonic weight. Our understanding of the molecular events governing this process has been hampered by the difficulty in accessing them in vivo under natural conditions. We hypothesize that the quality of the mature oocyte is regulated by crosstalk between the oocyte and the somatic microenvironment during extended storage prior to ovulation. By combining temporal profiling and tissue‐specific functional analyzes in *Drosophila*, we characterize a spatiotemporal crosstalk between the oocyte and the granulosa cells that governs preovulatory follicle aging in vivo. Preovulatory follicle aging is characterized by two distinct phases—early oocyte protective and late degenerative phases. The degenerative phase involves a positive feedback loop between oocyte mitochondrial dysfunction mediated by a mitochondrial‐localized microprotein PIGBOS, and granulosa cell functional decline through a circular RNA *circdlg1*. Activation of the feedback loop is suppressed by germline Sestrin during the early phase. Our findings highlight that natural preovulatory follicle aging in vivo is governed by a mechanism that represses an oocyte‐degenerative positive feedback loop between oocyte and granulosa cells.

## Graphical abstract

Preovulatory follicle aging is characterized by two distinct phases—early oocyte protective and late degenerative phases. The degenerative phase involves a positive feedback loop between oocyte mitochondrial dysfunction mediated by a mitochondrial‐localized microprotein PIGBOS, and granulosa cell functional decline through a circular RNA *circdlg1*. Activation of the feedback loop is suppressed by germline Sestrin during the early phase. Thus, preovulatory follicle aging is governed by a mechanism that represses an oocyte‐degenerative positive feedback loop between oocyte and granulosa cells.

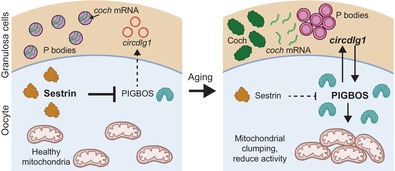

## 1 Introduction

Reproductive aging is the progressive decline in fertility with age (Dong et al. 2023). It comprises a branch of reproductive sciences that has diverse implications for human health, livestock farming, conservation of endangered species and pest control. In mammals, aging is associated with a decrease in ovarian reserve and oocyte quality. The ovarian reserve is defined as the fixed number of primordial follicles an animal is born with, which decreases with age (Gleicher et al. 2011; Yan et al. 2024). The quality of oocytes, on the other hand, depends on factors such as mitochondrial health, proteostasis, maternal RNAs and proteins and spindle morphology (Bomba‐Warczak et al. 2023; Cheng et al. 2022; Cox et al. 2021; Giaccari et al. 2024; Greenblatt et al. 2019; Harasimov et al. 2024; Jentoft et al. 2023; Pek 2024).

A less well‐understood aspect is the process of preovulatory follicle aging—the period between the formation and ovulation of a mature follicle. As opposed to chronological or organismal aging, preovulatory follicle aging leads to oocyte quality decline during the extended storage in the ovary prior to ovulation. It is known that delayed ovulation can lead to “over ripeness” of oocytes that are associated with chromosomal abnormalities and developmental defects such as decreased implantation, increased malformation and mortality and lower embryonic weight (Bittner et al. 2011; Butcher et al. 1969; Butcher and Fugo 1967; Fugo and Butcher 1966; Mikamo 1968; Peluso et al. 1980; Smits et al. 1995). While earlier studies had suggested preovulatory aging is linked to changes in mRNA polyadenylation, little is known about the molecular pathways governing this process (Dankert et al. 2014; Demond et al. 2016; Peluso and Butcher 1974).

One reason for the lack of advance in understanding preovulatory follicle aging is the difficulty in studying this process in vivo in mammals. Most studies relied on the use of drugs or hormones to delay ovulation, making it difficult to reveal the natural process of preovulatory follicle aging—mechanisms of oocyte selection and atresia and the impact on subsequent fertilization and embryonic development.

Insects offer an ideal system to study preovulatory follicle aging. They have evolved mechanisms to store mature oocytes to ensure survival in their natural habitats. Studies in *Drosophila melanogaster* (fruit fly) and *Aedes aegypti* (mosquito) have begun to reveal oocyte intrinsic and extrinsic mechanisms in preovulatory follicle aging (Greenblatt et al. 2019; Greenblatt and Spradling 2018; Venkataraman et al. 2023). However, the spatiotemporal coordination between oocytes and the microenvironment in preovulatory follicles during aging has not been reported. Moreover, bidirectional communication between the oocyte and the surrounding support cells is conserved from *Drosophila* to mammals including humans (Doherty et al. 2022). In both systems, oocytes receive signals from the supporting germline nurse cells and the somatic granulosa cells (also known as follicle cells in *Drosophila*) (Doherty et al. 2022; Lei and Spradling 2016; Niu and Spradling 2022; Spradling et al. 2022; Vachias et al. 2025; Wang 2025; Wang, Huang, et al. 2024). As mature follicles only contain oocytes without any nurse cells, we focus on the role of granulosa cells in regulating oocyte quality. In both *Drosophila* and mouse, granulosa cells are known to maintain oocyte health by transferring essential hormones, metabolites and RNA (Vachias et al. 2025; Wang 2025), and a decline in granulosa cell function may contribute to poorer oocyte quality during aging (Wang 2025). A recent study in mouse had shown that “young” granulosa cells can rejuvenate aged oocytes; however, the molecular mechanisms contributing to granulosa cell aging are unclear (Wang, Huang, et al. 2024). Understanding the molecular mechanisms that contribute to granulosa cell functional decline during aging will assist in developing novel cellular therapies in vivo.

**FIGURE 1:**
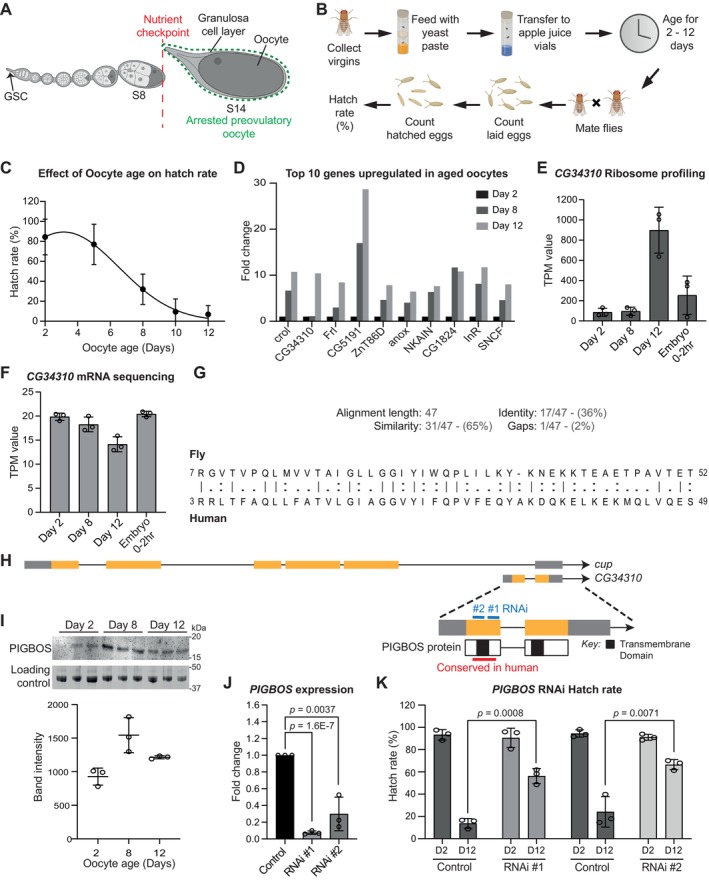
Oocyte PIGBOS promotes oocyte degeneration during aging. (A) *Drosophila* oogenesis starting from germline stem cell (GSC) stage to Stage 14 (S14) with the nutrient checkpoint occurring at Stage 8 (S8). Stage 14 is determined as the arrested preovulatory oocyte. (B) A schematic diagram of the process of aging *Drosophila* preovulatory follicles for 2–12 days in the absence of protein source (yeast) to determine the hatch rate. (C) The effects of aging on oocyte quality in control *y w* flies as determined by hatch rates. Data are presented as mean values ± SD. *n* = 24 (Day 2), 21 (Day 5), 18 (Day 8), 25 (Day 10, Day 12) flies. (D) Top 10 upregulated genes in aged (Day 12) oocytes identified from Greenblatt et al. (2019) ribosome profiling data. These genes show 6‐fold increase in expression between Day 2 and Day 12 with *p*‐value below 0.05. (E,F) Ribosome profiling and mRNA sequencing results for *CG34310* (*PIGBOS*) on Day 2, 8 and 12 oocytes and 0–2 h embryos obtained from Greenblatt et al. (2019). Data are presented as mean values ± SD with *n* = 3 biological replicates for all conditions. (G) Protein sequence alignment between *Drosophila* PIGBOS and human PIGBOS1 with a similarity of 65%. Data obtained using DRSC Integrative Ortholog Prediction Tool (DIOPT). (H) Gene locus for *CG34310* (*PIGBOS*) that is part of *cup* 3′ UTR. The area of *CG34310* that is conserved in human is annotated in red, while the *PIGBOS* RNAi #1 and #2 target areas are annotated in blue. The transmembrane domains of PIGBOS protein shown are aligned to the gene locus, with one of the domains conserved in humans. These domains are predicted using TOPCONS webserver. (I) Upregulation of PIGBOS expression during aging in control *y w* oocytes. Graph indicates the relative levels of PIGBOS normalized to the loading control (total protein stained with coomassie blue). Data are presented as mean values ± SD with *n* = 3 biological replicates. (J) The relative expression of *PIGBOS* in *PIGBOS* RNAi #1 and #2 compared to control (*Oregon R*). Data are mean values ± SD with *n* = 3 biological replicates. *p* values were calculated using two‐tailed Student's *t*‐test. (K) A universal drop of Day 12 (D12) hatch rates when compared to Day 2 (D2) with improvements in hatch rates in Day 12 *vasa>PIGBOS* RNAi flies as compared to Day 12 sibling controls using 2 independent RNAi transgenes. Data are mean values ± SD with *n* = 3 biological replicates. *p* values were only calculated for the hatch rates between Day 12 oocytes comparing RNAi and their respective control using two‐tailed Student's *t*‐test.

We reveal the cellular and molecular mechanisms governing the natural aging process of preovulatory follicles in *Drosophila*, where mature oocytes are encapsulated by a layer of post‐mitotic somatic granulosa cells (Figure 1A). Oocyte aging is characterized by an initial gradual decline in quality, which is followed by a drastic drop during the second phase. A mitochondrial‐localized protein PIGBOS promotes oocyte mitochondrial clumping and dysfunction. This process in turn triggers the upregulation of a circular RNA (circRNA) *circdlg1* in the granulosa cells, which seeds the formation of P‐bodies leading to functional decline. Interestingly, oocyte PIGBOS and granulosa cell *circdlg1* form a positive feedback loop by mutually reinforcing each other's expression leading to oocyte degeneration. Activation of the positive feedback loop is suppressed by Sestrin (Sesn) during the initial oocyte‐protective phase of aging. Thus, preovulatory follicle aging is governed by a mechanism that represses an oocyte degenerative positive feedback loop between oocyte and granulosa cells.

## 2 Results

### 2.1 PIGBOS Promotes Oocyte Degeneration During Aging

We adapted a previously established protocol to examine the effects of prolonged storage or aging of preovulatory mature stage 14 follicles in *Drosophila* (Figure 1A,B) (Greenblatt et al. 2019; Greenblatt and Spradling 2018). We observed a gradual decrease in hatch rates as preovulatory follicles age, similar to what was reported previously (Greenblatt et al. 2019). At 25°C, our results show a faster decrease in hatch rates of < 10% on day 12 (Figure 1C), as compared to ~50% on day 12 as reported before. This is likely due to differences in environmental factors such as humidity or the differences in food recipes for aging oocytes. Ovulation is triggered by the ecdysone steroid hormone signaling pathway in the granulosa cells of the mature follicles (Knapp and Sun 2017). We delayed ovulation by expressing a dominant negative form of ecdysone receptor in the granulosa cells as previously reported and observed accelerated aging at day 8 (Figure S1). Together, these data indicate that prolonged storage of mature follicles leads to oocyte quality decline.

Previous ribosome profiling of preovulatory oocytes during aging identified translation downregulation as a major effect contributing to defects in meiotic spindle morphology and chromosome segregation (Greenblatt et al. 2019). While a general downregulation of translation may be due to a deterioration of the translation machinery during aging, accumulation of some defective or regulatory proteins may also lead to disruption of normal cellular functions. To identify potential genes that play an active role in regulating oocyte quality during aging, we re‐examined previously published ribosome profiling datasets to instead focus on genes that are translationally upregulated in aged poor‐quality oocytes (Greenblatt et al. 2019). Among the genes that show translation upregulation, *CG34310* caught our attention because its translation efficiency, but not mRNA abundance, was significantly increased only in day 12 oocytes (Figure 1D–F). *CG34310* is an uncharacterized gene in *Drosophila* predicted to encode a 129 amino acid protein, with a mammalian homolog called PIGBOS1 (Figure 1G,H) (Hu et al. 2011). Mammalian PIGBOS1 encodes a microprotein that localizes to the mitochondria and regulates endoplasmic reticulum (ER) stress (Chu et al. 2019). We generated a specific antibody against *Drosophila CG34310* (hereafter known as *PIGBOS*) and found that its abundance was increased in day 8 and 12 stage 14 oocytes (Figures 1I and S2A), consistent with an upregulation of PIGBOS in poor‐quality oocytes during aging.

*PIGBOS* is a transcriptionally independent locus embedded within the *cup* locus (Figure 1H). It contains two exons where the unique first exon contains sequences that are conserved in mammalian PIGBOS1, whereas exon 2 overlaps with *cup*'s last exon. To examine the function of PIGBOS, we designed 2 short‐hairpin (sh)RNAs that target different regions of exon 1 (Figure 1H). Germline‐specific expression of the shRNAs led to robust knockdown of *PIGBOS* mRNA expression, but not *cup* mRNA, in the ovaries (Figures 1J and S2B). Importantly, germline *PIGBOS* RNAi led to significant improvement of hatch rates in day 12 oocytes (Figures 1K and S2C), suggesting that PIGBOS is oocyte degenerative during aging. Moreover, overexpression of PIGBOS CDS rescued the RNAi hatch rate (Figure S3).

### 2.2 PIGBOS Forms Mitochondrial‐Associated Bodies

**FIGURE 2:**
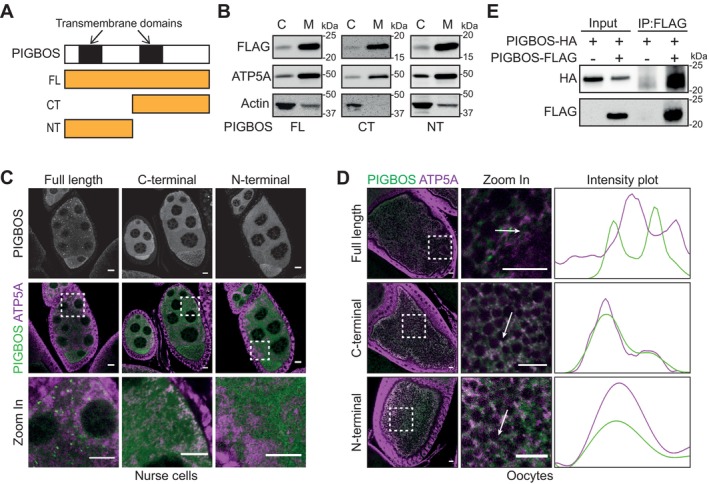
PIGBOS forms mitochondrial‐associated bodies. (A) PIGBOS protein with the transmembrane domains highlighted in black, aligned with Full‐length (FL), C‐terminal (CT) and N‐terminal (NT) versions of the protein generated for experiments. (B) Enrichment of PIGBOS‐FLAG proteins (FL, CT, NT) in mitochondrial (M) fractions but not in cytosol (C) fractions. The mitochondrial and cytosol fraction are identified using ATP5a and Actin, respectively. (C) Immunostaining of stage 8/9 egg chambers shows punctate distribution pattern of PIGBOS FL, but not CT and NT (PIGBOS: Green and ATP5a: Magenta). Zoomed in region are bound by dotted squares. Scale bar = 10 μm. (D) Immunostaining of oocytes in stage 10 egg chambers shows punctate distribution pattern of PIGBOS FL, but not CT and NT (PIGBOS: Green and ATP5a: Magenta). The intensity plots show the localization patterns between PIGBOS FL, CT and NT with ATP5a. Zoomed in region are bounded by dotted square. Scale bar = 10 μm. (E) Co‐immunoprecipitation between PIGBOS FL tagged with FLAG and PIGBOS FL tagged with HA.

In silico analysis indicates that PIGBOS contains 2 transmembrane domains and is predicted to localize to the mitochondrial outer membrane (Figure S4A). The first transmembrane domain is conserved in mammalian PIGBOS1, while the second one is unique to *Drosophila* (Figure 1H). We generated FLAG‐tagged full‐length (FL) and truncated (C‐terminus CT and N‐terminus NT) versions of PIGBOS and examined their ability to localize to mitochondria (Figure 2A). Germline expression of PIGBOS‐FLAG followed by mitochondrial fractionation of defolliculated stage 14 oocytes showed that FL, CT and NT versions localized to mitochondria (Figure 2B), suggesting that truncations did not affect mitochondrial localization.

Immunostaining of FLAG‐PIGBOS showed some intriguing localization patterns in ovaries. In nurse cells, PIGBOS‐FL formed cytoplasmic puncta‐like structures that decorate mitochondria (marked by ATP5A) (Figure 2C). Such puncta were not seen in PIGBOS‐CT and NT where they appeared diffuse in the cytoplasm with some colocalization with mitochondria (Figure 2C). Such localization patterns were also observed in oocytes of stage 10 follicles where PIGBOS‐FL localized as puncta that are closely associated with mitochondria (Figure 2D). Removal of either N or C terminal sequences in PIGBOS‐CT or NT respectively abolished the formation of puncta, resulting in colocalization with mitochondria (Figure 2D). Overall, our experiments show that PIGBOS localizes to mitochondria, which is consistent with its mammalian counterparts.

The ability to self‐assemble into higher‐order protein complexes is a hallmark of many mitochondrial outer membrane proteins and proteins that form cellular bodies (Boke et al. 2016; Bose et al. 2022, 2024). By expressing both FLAG‐ and HA‐tagged PIGBOS in S2 cells, we found that PIGBOS‐FL could interact as a complex (Figure 2E). We observed that this interaction could only occur between PIGBOS‐FL as truncations in either CT or NT abolished their interactions (Figure S4B,C). These observations correlate strongly with the ability of PIGBOS to form cytoplasmic bodies (Figure 2C,D), suggesting that such complex formation is needed for puncta formation.

### 2.3 PIGBOS Promotes Mitochondrial Clumping and Dysfunction

**FIGURE 3:**
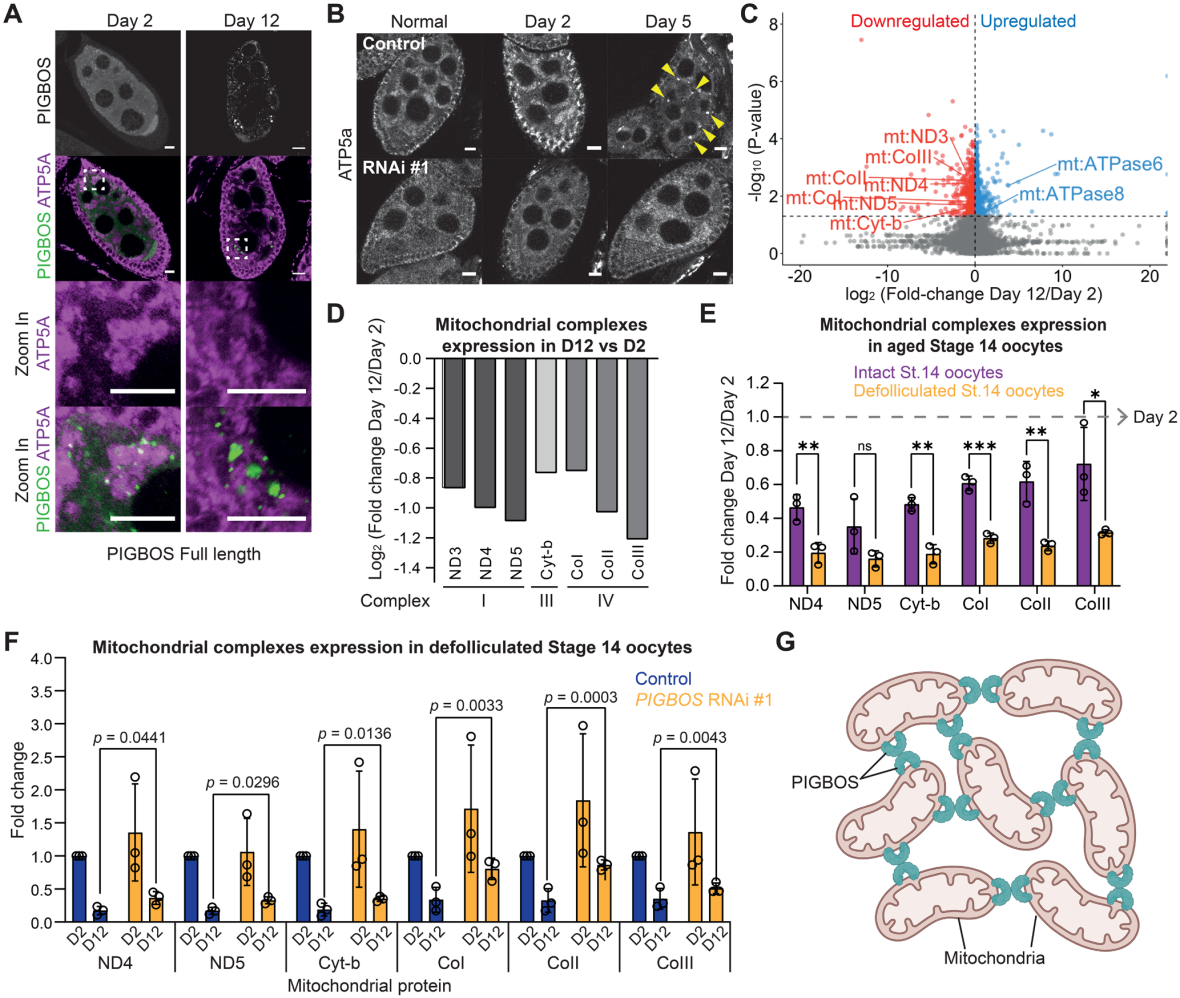
PIGBOS promotes mitochondrial clumping and dysfunction. (A) Immunostaining of stage 8/9 egg chambers aged for 2 and 12 days with PIGBOS (Green) and ATP5a (Magenta). PIGBOS foci decorate mitochondrial clumps and become bigger during aging. Zoomed in region are bound by dotted squares with ATP5a single channel and ATP5a with PIGBOS merged channel shown. Scale bar = 10 μm. (B) Immunostaining shows the clumping of mitochondria (ATP5a, arrowheads) stage 8/9 egg chambers of control (*Oregon R*) but not in *vasa‐Gal4>PIGBOS* RNAi #1 when aged for 2 and 5 days. Scale bar = 10 μm. (C) RNA sequencing data of oocytes aged for 2 and 12 days with downregulated (red) and upregulated (blue) mitochondrial genes indicated. Only genes with *p* < 0.05 are colored. *N* = 3 biological replicates. (D) Downregulated mitochondrial genes from RNA sequencing data are shown with their respective mitochondrial complexes indicated and relative expression in Day 12 (D12) compared to Day 2 (D2). (E) RT‐qPCR showing the relative expression of mitochondrial complex I, III and IV genes in control Stage 14 oocytes, with and without follicle cells, aged for 2 and 12 days. Data are presented as mean values ± SD. ns = not significant, *p < 0.05, **p < 0.01 and ***p < 0.001. Student's *T*‐test. *N* = 3 biological replicates. (F) RT‐qPCR showing the relative expression of mitochondrial complexes genes in control and *PIGBOS* RNAi #1 defolliculated Stage 14 oocytes aged for 2 days (D2) and 12 days (D12). Data are presented as mean values ± SD with *n* = 3 biological replicates. The exact *p*‐value shown was obtained using Student's *T*‐test. (G) Proposed model of the interaction between PIGBOS and mitochondria in aged oocytes.

Under nutrient‐deprived conditions, stage 8/9 follicles are arrested and mitochondria clump around the nurse cell nuclei (Cox and Spradling 2009; Ng et al. 2024b). Generally, clumping of mitochondria is induced by oxidative damage and mutations affecting mitochondrial functions, and is associated with unhealthy or damaged mitochondria (Cox and Spradling 2009; DeVorkin et al. 2014; Sen et al. 2013, 2015; Sheard and Cox 2021). To understand the role of PIGBOS in regulating mitochondrial functions, we examined the localization of FLAG‐PIGBOS in stage 8/9 follicles during arrest. At day 2, PIGBOS puncta were seen decorating the mitochondrial clumps (Figure 3A). These puncta became more intense and bigger under prolonged arrest as seen in day 12 follicles (Figure 3A), suggesting that they may maintain or enhance mitochondrial clumping during arrest. In support of this hypothesis, mitochondrial clumping was severely inhibited in *PIGBOS* RNAi follicles under arrest (Figure 3B). Thus, PIGBOS is needed for mitochondrial clumping in arrested follicles.

To examine the changes in gene expression during preovulatory follicle aging, we profiled total RNA from day 2 and 12 follicles without defolliculation. Consistent with a reduction in mitochondrial function or activity, the expression of several mitochondrial‐encoded RNAs that constitute Complexes I, III and IV was downregulated (Figure 3C). These include NADH–ubiquinone oxidoreductase chain 3 (ND3), ND4 and ND5 in Complex I, Cytochrome b (Cyt‐b) in Complex III and Cytochrome c oxidase subunit I (CoI), CoII and CoIII in Complex IV (Figure 3D). The downregulation of these mitochondrial genes was further confirmed by quantitative RT‐PCR in both intact follicles and defolliculated stage 14 oocytes (Figure 3E). Since somatic granulosa cells may also contribute to the regulation of mitochondrial genes, we compared the extent of change between intact follicles and defolliculated stage 14 oocytes. The magnitude of downregulation was significantly greater in defolliculated oocytes than in intact follicles (Figure 3E), indicating that mitochondrial activity in oocytes is indeed perturbed during aging. In addition, the downregulation of these mitochondrial‐encoded genes was partially alleviated in *PIGBOS* RNAi oocytes, suggesting that PIGBOS mediates the suppression of mitochondrial activity during aging (Figure 3F). Together, our experiments suggest that during aging, PIGBOS promotes clumping of mitochondria (Figure 3G) and suppresses mitochondrial activity leading to a reduction in preovulatory oocyte quality.

### 2.4 Granulosa Cell *circdlg1* Promotes Oocyte Degeneration During Aging

**FIGURE 4:**
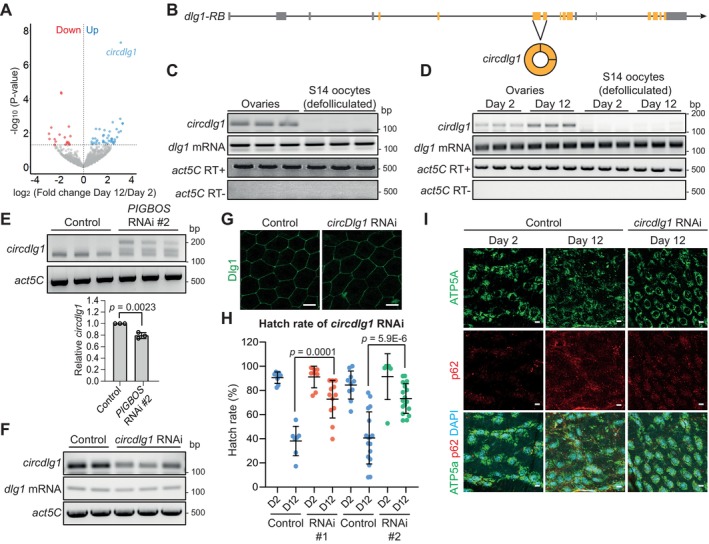
Granulosa cell *circdlg1* promotes oocyte degeneration during aging. (A) Volcano plot depicting differentially expressed circRNAs between day 2 and 12 stage 14 oocytes. *N* = 3 biological replicates. (B) The *dlg1* locus showing the circRNA‐producing exons generated by back‐splicing. (C) RT‐PCR showing the presence of *circdlg1* in whole ovaries but not in defolliculated stage 14 (S14) oocytes, indicating their localization in somatic cells (follicle cells) instead of germ cells (oocytes). (D) RT‐PCR showing the upregulation of *circdlg1* in day 12 ovaries with no detection on day 2 and 12 defolliculated S14 oocytes. (E) RT‐PCR showing the reduction of mature *circdlg1* in *vasa>PIGBOS* RNAi ovaries. Transcripts of higher molecular weights, depicting misprocessed *circdlg1* intermediates were seen in *vasa>PIGBOS* RNAi ovaries. (F,G) RT‐PCR and immunostaining showing specific downregulation of *circdlg1* but not *dlg1* mRNA and protein in *GMR47A04>circdlg1* RNAi ovaries and granulosa cells. Scale bar = 20 μm. (H) Improvements in hatch rates in day 12 (D12) *GMR47A04>circdlg1* RNAi flies as compared to D12 controls using 2 independent RNAi transgenes. Hatch rates in day 2 (D2) do not show any significant changes, hence the *p‐*value is not stated. Data are presented as mean values ± SD with *n* = 3 biological replicates. The exact *p*‐value shown was obtained using Student's *T*‐test. (I) Immunostaining showing fragmented mitochondria (ATP5A, green) and upregulation of p62 puncta (red) in control day 12 as compared to day 2 granulosa cells. This phenotype was rescued in day 12 *GMR47A04>circdlg1* RNAi granulosa cells. Scale bar = 10 μm.

To further explore the mechanisms of preovulatory follicle aging, we switched our attention to circular RNAs (circRNAs), which belong to a distinct class of RNAs with covalently linked ends and are generally produced by back‐splicing (Chen 2020). Due to the absence of 5′ and 3′ ends, circRNAs are stable and accumulate in post‐mitotic cells such as neurons. Some circRNAs have been shown to accumulate during aging and function in modulating lifespan and mediating age‐related diseases (Kim et al. 2021; Weigelt et al. 2020; Westholm et al. 2014). We profiled circRNAs from intact day 2 and 12 stage 14 follicles and identified a total of ~2748 circRNAs. The majority of them did not change their abundance during aging, consistent with their highly stable properties. Among them, 18 circRNAs were downregulated and 43 were upregulated in day 12 follicles (Figure 4A). We identified a circRNA from the *dlg1* locus (hereafter known as *circdlg1*) that is significantly upregulated in day 12 follicles (Figures 4A,B and S5A). *circdlg1* was previously identified in *Drosophila* brain but its function has not been reported (Westholm et al. 2014).

Oocytes are transcriptionally quiescent cells, suggesting that the upregulation of *circdlg1* might be coming from the somatic granulosa cells. This was confirmed by defolliculation of stage 14 oocytes, which resulted in a total loss of *circdlg1* (Figure 4C), indicating that *circdlg1* is highly expressed in granulosa cells. We further confirmed the upregulation of *circdlg1* in aged follicles, which was not detected after defolliculation (Figure 4D).

We observed that the abundance of *circdlg1* was reduced in germline *PIGBOS* RNAi ovaries, concomitant with the appearance of mis‐processed *circdlg1* of higher molecular weights (Figures 4E and S5B), placing PIGBOS upstream of *circdlg1*. The observation suggested that *circdlg1* may promote oocyte degeneration during aging. We generated 2 independent shRNA lines that specifically target the back‐spliced junction of *circdlg1*. Granulosa cell‐specific expression of both shRNAs led to down‐regulation of *circdlg1* but not its cognate *dlg1* mRNA and Dlg1 protein, indicating a specific knockdown of circRNA (Figures 4F,G and S5C). Knockdown of *circdlg1* in the granulosa cells also resulted in improvements in hatch rates of day 12 oocytes (Figure 4H), supporting a role for *circdlg1* in promoting oocyte degeneration during aging.

Day 12 granulosa cells exhibited signs of mitochondrial fragmentation and proteostasis (as shown by an increase in p62 puncta) (Figure 4I). This phenotype was suppressed in *circdlg1* RNAi (Figure 4I), suggesting that *circdlg1* mediates granulosa cell functional decline during aging.

### 2.5 *circdlg1* Promotes the Formation of P‐Bodies During Aging

**FIGURE 5:**
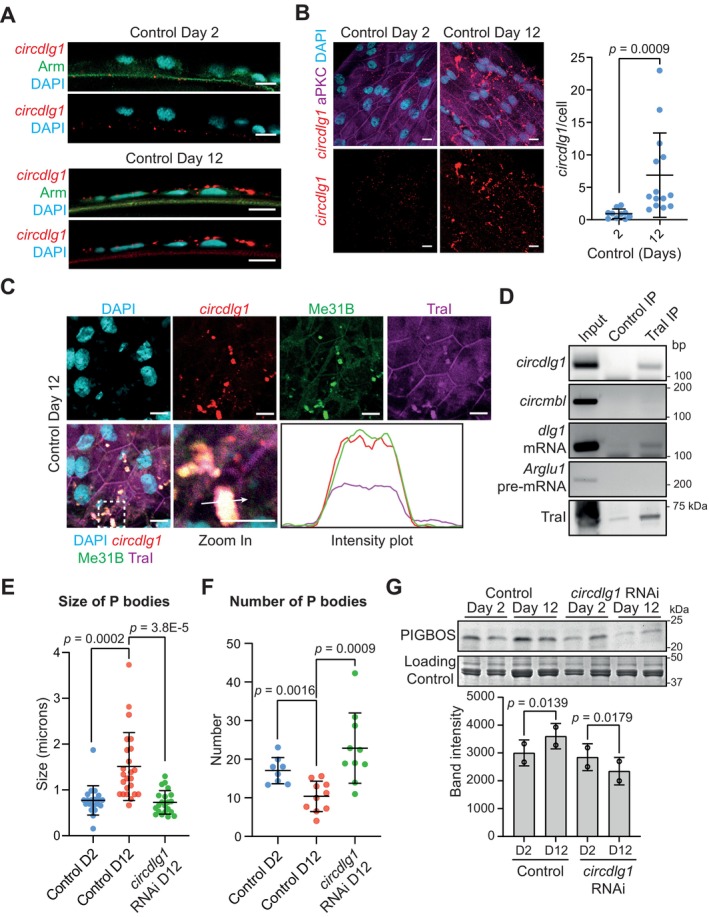
*circdlg1* promotes the formation of P‐bodies during aging. (A) Immunostaining and smFISH showing localization of *circdlg1* (red) on the apical surface (Arm, green) of day 2 granulosa cells. This localization is disrupted on day 12 granulosa cells, where *circdlg1* localized away from the apical surface. Scale bar = 10 μm. (B) Increase in *circdlg1* signals (red) in day 12 granulosa cells. Cell boundaries are marked by aPKC (magenta). Scale bar = 10uM. Data are presented as mean values ± SD. The exact *p*‐value shown was obtained using Student's *T*‐test. (C) Colocalization of *circdlg1* (red) with P‐body markers Me31B (green) and Tral (magenta) in day 12 granulosa cells. The intensity plot shows the co‐localization of *circdlg1*, Me31B and TraI. Zoomed in region are bound by dotted square. Scale bar = 10 μm. (D) Immunoprecipitation of Tral in day 12 ovaries showed specific binding to *circdlg1* but not *circmbl*. (E,F) The size and number of P‐bodies in granulosa cells increase and decrease, respectively during aging, but was rescued in *GMR47A04>circdlg1* RNAi (D2 = Day 2; D12 = Day 12). Data are presented as mean values ± SD with *n* = 3 biological replicates. The exact *p*‐value shown was obtained using Student's *T*‐test. (G) PIGBOS expression is upregulated during aging in control ovaries but not in *GMR47A04>circdlg1* RNAi (D2 = Day 2; D12 = Day 12). Data are presented as mean values ± SD with *n* = 2 biological replicates. The exact *p*‐value shown was obtained using Student's *T*‐test.

Visualization of *circdlg1* using single molecule fluorescent in situ hybridization (smFISH) showed a significant increase in *circdlg1* in day 12 granulosa cells (Figure 5A,B), consistent with RNA sequencing and RT‐PCR data (Figure 4A–D). Closer inspection revealed that in day 2 granulosa cells, *circdlg1* localized on the apical surface as marked by Armadillo (Figure 5A). This polarized localization was disrupted in day 12 granulosa cells, where *circdlg1* localized away from the apical surface appearing as larger punctate‐like structures (Figure 5A).

The *Drosophila* aging brain is associated with the appearance of repressive P‐bodies (Pushpalatha et al. 2022). In day 12 granulosa cells, *circdlg1* puncta colocalized with P‐body markers maternal expression at 31B (Me31B) and Trailer Hitch (Tral) (Figure 5C), suggesting that *circdlg1* may interact with the P‐body. We confirmed a specific interaction between *circdlg1* and Tral by performing immunoprecipitation of Tral using day 12 follicle lysate (Figure 5D).

Since noncoding RNAs can seed the formation of cellular bodies, we looked for evidence of whether *circdlg1* may promote the formation of P‐bodies during aging (Bose et al. 2024; Chan and Pek 2022). During aging, the number of P‐bodies decreased concomitant with an increase in size (Figure 5E,F), suggesting that P‐bodies coalesce. This was accompanied by an increase in *circdlg1* expression and *circdlg1* binding to Tral (Figure 5A–D). Indeed, the knockdown of *circdlg1* rescued the age‐dependent effect on P‐body size and number (Figure 5E,F), providing evidence that *circdlg1* promotes the coalescence of P‐bodies during aging.

Since *circdlg1* RNAi resulted in an improvement in oocyte quality during aging, we asked if *circdlg1* regulates PIGBOS during aging. Interestingly, the abundance of PIGBOS was not upregulated in *circdlg1* RNAi follicles during aging (Figure 5G), suggesting that *circdlg1* promotes age‐dependent accumulation of PIGBOS.

### 2.6 Age‐Dependent Upregulation of Coch Contributes to Oocyte Quality Decline

**FIGURE 6:**
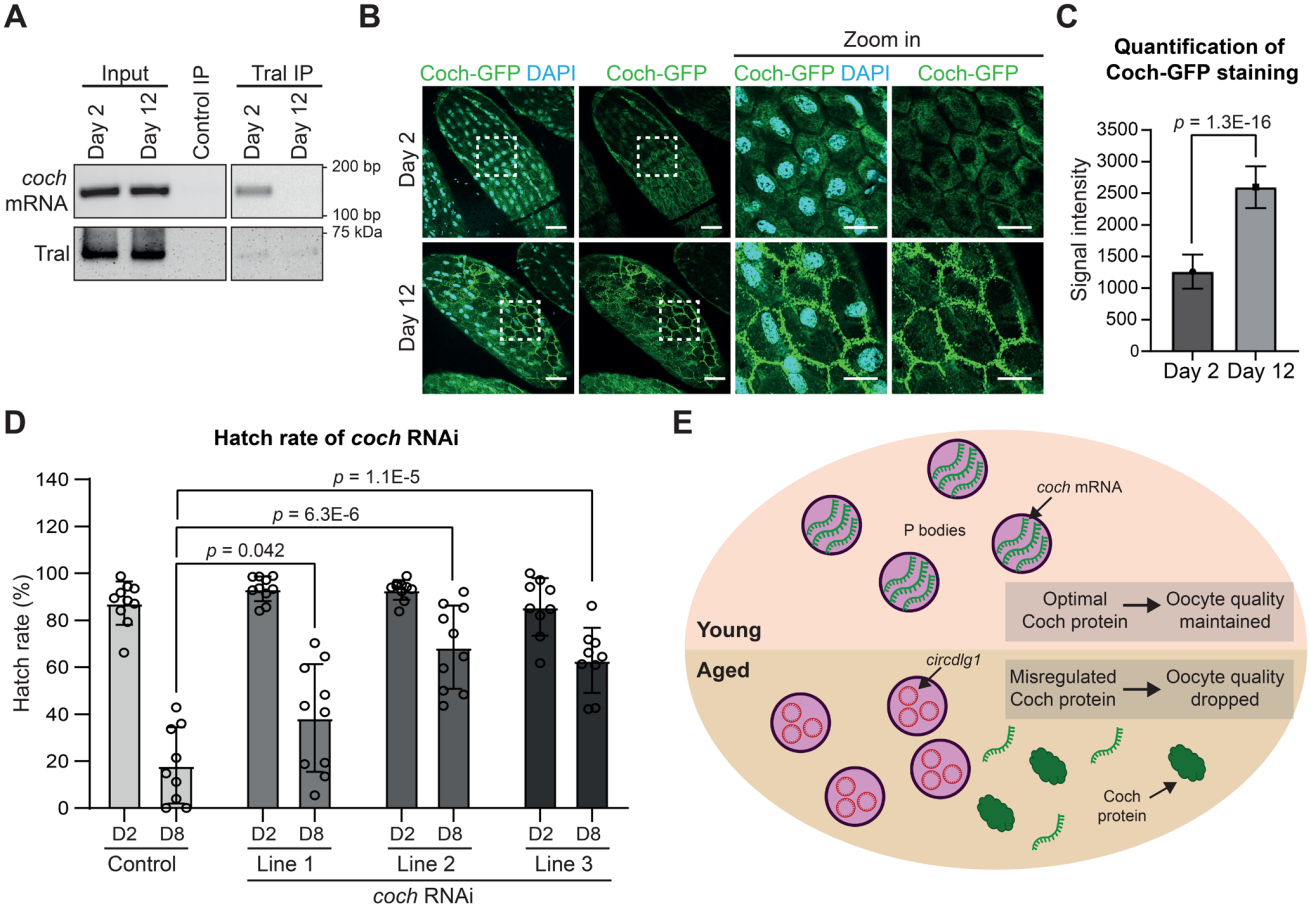
Age‐dependent upregulation of Coch contributes to oocyte quality decline. (A) Immunoprecipitation of TraI in Day 2 and Day 12 ovaries shows binding with *coch* mRNA at Day 2 but not in Day 12. (B) Immunostaining of the granulosa cells shows increased Coch‐GFP expression in Day 12. Zoomed in regions are bounded by dotted squares. Scale bar = 50 μm for whole oocytes, 10 μm for zoomed in regions. (C) Quantification of Coch‐GFP intensity as shown in (B). Data are presented as mean values ± SD. The exact *p*‐value shown was obtained using Student's *T*‐test. (D) Improvements of hatch rates in day 8 in *GMR47A04*>*coch* RNAi flies as compared to sibling control. (D2 = Day 2; D8 = Day 8). Line 1: TRiP.GLV21061, Line 2: TRiP.GL01304, Line 3: TRiP.HMJ22481. Data are presented as mean values ± SD. The exact *p*‐value shown was obtained using Student's *T*‐test. (E) A model depicting the release of *coch* mRNA from P‐bodies during aging, resulting in mis‐regulation of Coch protein translation. This promotes oocyte quality decline during preovulatory follicle aging.

To better understand the function of P‐body during preovulatory follicle aging, we examined *cochonnet* (*coch*) mRNA (orthologous to several human genes encoding solute carrier family members SLC36A1, SLC36A2 and SLC36A4). In *Drosophila*, *coch* has been recently shown to be important for somatic follicle cell functions and binds to translating ribosomes (Vachias et al. 2025). We found that *coch* mRNA is bound to Tral at day 2 follicles (Figure 6A), suggesting that translation of *coch* mRNA is regulated by P‐bodies. Interestingly, binding of *coch* mRNA to Tral is diminished in day 12 follicles (Figure 6A). This observation suggests that *coch* mRNA is released from the P‐bodies during aging. Consistent with this observation, we found significant upregulation of Coch protein in day 12 granulosa cells (Figure 6B,C), supporting the idea that translation repression was alleviated. To decipher the biological consequence of Coch upregulation during aging, we knocked down *coch* mRNA using 3 independent RNAi lines, and observed an improvement in hatch rates at day 8 (Figure 6D), in line with a delay in preovulatory follicle aging. Taken together, these data suggest that aging leads to dysregulation of *coch* mRNA translation by P‐bodies contributing to a decline in oocyte quality.

### 2.7 Sesn is Oocyte‐Protective by Suppressing PIGBOS and *circdlg1*

**FIGURE 7:**
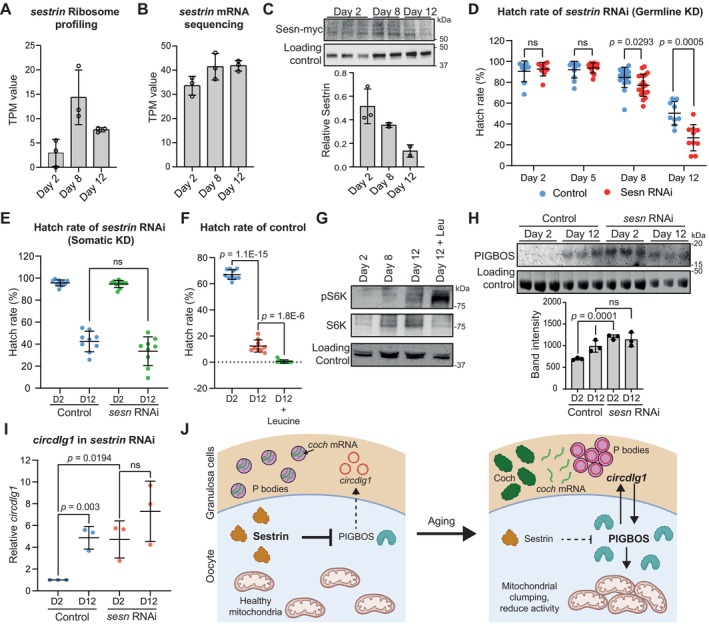
Sesn is oocyte‐protective by suppressing PIGBOS and *circdlg1*. (A,B) Downregulation of translation of *sesn* from day 8 to 12 independent of mRNA levels in stage 14 oocytes. (C) Decrease in Sesn‐myc endogenous expression during oocyte aging. (D,E) Germline *MTD>sesn* RNAi, but not somatic granulosa cell *GMR47A04>sesn* RNAi, leads to lower hatch rates during aging. Data are presented as mean values ± SD. The exact *p*‐value shown was obtained using Student's *T*‐test. ns = not significant. (F) Hatch rates of control *y w* flies during aging are further exacerbated by feeding with Leucine from day 8 onwards. The exact *p*‐value shown was obtained using Student's *T*‐test. (G) Western blots showing the levels of pS6K and S6K in the indicated conditions in *y w* ovaries. Actin was used as a loading control. (H) PIGBOS expression is upregulated in day 2 *MTD>sesn* RNAi ovaries and is comparable to day 12 *MTD>+* controls. (D2 = Day 2; D12 = Day 12). The exact *p*‐value shown was obtained using Student's *T*‐test. ns = not significant. (I) *circdlg1* expression is upregulated in day 2 (D2) *MTD>sesn* RNAi ovaries and is comparable to day 12 (D12) *MTD>+* controls. Data are presented as mean values ± SD. *N* = 3 biological replicates. The exact *p*‐value shown was obtained using Student's *T*‐test. ns = not significant. (J) Model depicting the events happening in preovulatory oocyte and granulosa cell during aging.

The mutual regulation between PIGBOS and *circdlg1* suggests a positive feedback loop between PIGBOS in oocytes and *circdlg1* in granulosa cells, which may accelerate oocyte degeneration once triggered during aging. This is in agreement with observations that oocyte quality is maintained during early aging and drops drastically thereafter (Figure 1C), suggesting an early oocyte‐protective mechanism in suppressing the PIGBOS‐*circdlg1* feedback loop. Revisiting previously reported ribosome profiling data revealed a gene *sestrin* (*sesn*) that is translationally upregulated during early aging and drops thereafter, correlating with oocyte quality (Figure 7A,B). Sesn plays diverse roles in stress response pathways and age‐related decline in various animals; however its role in oocyte aging is unknown (Lee et al. 2010; Lu et al. 2021).

By monitoring endogenous Sesn expression using a myc‐tag knock‐in strain, we observed a correlation between Sesn abundance in stage 14 oocyte and oocyte quality (Figure 7C). Germline *sesn* RNAi resulted in a premature preovulatory follicle aging from day 8 onwards (Figure 7D). In contrast, no significant decrease was observed when *sesn* was knocked down in granulosa cells (Figure 7E), suggesting an oocyte‐protective role of *sesn* during aging. Previous reports had shown that the activity of Sesn is suppressed by binding to Leucine, an essential amino acid (Cangelosi et al. 2022; Gu et al. 2022; Lu et al. 2021). We fed wild‐type flies with Leucine starting from day 8 of aging and observed that this exacerbated the decline in oocyte quality at day 12 (Figure 7F), supporting a role for *sesn* in maintaining oocyte quality during aging. Consistent with a role of Sesn in suppressing the TOR pathway, the abundance of pS6K in ovaries was upregulated during aging and in Leucine‐fed flies (Figure 7G).

Further analyzes revealed that PIGBOS was upregulated in *sesn* RNAi day 2 follicles, comparable to the level of day 12 control follicles (Figure 7H), suggesting that *sesn* represses PIGBOS. Consistent with this observation, *circdlg1* expression was also increased in *sesn* RNAi day 2 follicles (Figure 7I). Together, these experiments suggest that *sesn* protects oocyte quality by suppressing the PIGBOS‐*circdlg1* feedback loop during aging (Figure 7J).

## 3 Discussion

### 3.1 Oocyte‐Granulosa Cell Crosstalk During Preovulatory Follicle Aging

Previous studies in insects had shown the involvement of oocyte intrinsic and extrinsic granulosa cell factors regulating preovulatory follicle aging. In flies, translation regulation during aging plays an important role in governing meiotic spindle morphology, subsequent chromosome segregation and embryonic neurogenesis (Greenblatt et al. 2019; Greenblatt and Spradling 2018). In contrast, studies in mosquitoes revealed the role of rapidly evolving genes functioning in granulosa cells for effective egg retention (Venkataraman et al. 2023). Our studies provide new insights into how oocyte mitochondria function in coordination with granulosa cell health during aging (Figure 7J). This model provides a framework for us to understand the cellular basis for preovulatory follicle aging.

In *Drosophila*, granulosa cells of preovulatory follicles function to control ovulation and pole plasm formation, providing further evidence of soma‐to‐oocyte communication (Deady et al. 2015; Mallart et al. 2024). Mechanisms in flies may be conserved in mammals as the somatic microenvironment also plays an important role in oocyte rejuvenation in culture (Wang, Huang, et al. 2024). A recent study in *Drosophila* showed that, like in mammals, somatic granulosa cells transfer important metabolites to the oocytes via gap junctions (Vachias et al. 2025).

Our study reveals that oocyte mitochondria signal to granulosa cells during aging. This is in agreement with a recent study in *Drosophila* showing that during oogenesis, germline cells signal to granulosa cells (or follicle cells) via reactive oxygen species (ROS) produced by germline mitochondria (Du et al. 2024). Such principles in mitochondrial dysfunction‐induced cellular crosstalk may be common in tissue aging as seen in the *Drosophila* brain (Byrns et al. 2024). Interestingly, in *Caenorhabditis elegans* piwi‐interacting RNAs (piRNAs) and hedgehog signaling from the germline regulate somatic aging, providing another possible mechanism for oocyte‐to‐soma communication (Shi and Murphy 2023; Zhou et al. 2024).

### 3.2 Biphasic Mode of Preovulatory Follicle Aging

Our study identifies preovulatory follicle aging occurs in two phases. The early period involves an oocyte‐protective mechanism by *sesn*, while the late phase is characterized by activation of an oocyte‐degenerative positive feedback loop. This phenomenon suggests that the timing of ovulation and oocyte retrieval is important for oocyte competence. Delayed ovulation has been linked to chromosomal abnormalities and developmental defects such as decreased implantation, increased malformation and motility and lower embryonic weight in vertebrates and humans (Bittner et al. 2011; Butcher et al. 1969; Butcher and Fugo 1967; Mikamo 1968; Smits et al. 1995). Clinically, women with endocrine irregularities may be more prone to fertility and embryonic issues (Smits et al. 1995). The ability to monitor follicle aging and assess egg quality with precision will improve the chances of spontaneous conception. Importantly, there is a dearth of biomarkers which can predict oocyte quality other than maternal age. Therefore, this work demonstrates that biomolecules unique to oocyte quality other than attributable by chronological aging maybe intrinsically involved in oocytes from women of all ages.

### 3.3 PIGBOS in Regulating Mitochondria Functions

In this study, we used nutrient‐deprivation to arrest stage 8/9 egg chambers to investigate the functions of PIGBOS in mitochondrial clumping. Downregulation of insulin signaling via nutrient‐deprivation is likely similar to the developmental downregulation of insulin signaling at stage 10–14 egg chambers when they enter quiescence (Greenblatt and Spradling 2018; Sieber et al. 2016).

We propose that PIGBOS functions like a molecular ‘glue’ that facilitates mitochondria clustering or clumping in arrested cells like oocytes (Figure 3G). Studies in mammalian cells showed that PIGBOS localizes on the outer mitochondrial membrane. We found that it localizes to the inter‐mitochondrial space as discrete bodies and is required for mitochondrial clumping in cells during arrest. The formation of inter‐mitochondrial bodies is associated with the ability of PIGBOS to interact in a complex, suggesting that PIGBOS inter‐molecular interactions regulate mitochondrial dynamics. The involvement of RNA‐mediated phase separation of PIGBOS is an intriguing area to explore in the future. Phase separation of membrane‐bound proteins may be a possible mode of regulation as seen in synaptic vesicle clustering and formation of Golgi ribbon (Song and Augustine 2023; Zhang and Seemann 2024). In *Drosophila* germline cells, a stable intron *sisR‐1* localizes to and promote mitochondrial clumping during arrest during aging (Ng et al. 2024b). It will be interesting to explore possible interactions between PIGBOS and *sisR‐1* in the formation of biomolecular condensates, which can be potentially used as a target for oocyte rejuvenation.

### 3.4 Regulation of PIGBOS and Mitochondria by Sesn

A study in mouse using single knockouts had shown that *Sesn1*, *2* and *3* are not required for female fertility even in aged 12‐month‐old mice (Wang, Chen, et al. 2024). However, it remains to be determined whether the 3 *Sesn* genes play redundant roles during oogenesis, and sesn has any functions in the context of preovulatory follicle aging, which are not examined in the study.

In mammals, SESN2 has been suggested to facilitate mitophagy (Kumar and Shaha 2018). We speculate that a similar mechanism may occur in *Drosophila* oocytes. During aging, downregulation of Sesn may lead to upregulation of the TOR pathway as shown by an upregulation of pS6K (Figure 7G) (Gu et al. 2022). This may in turn suppress mitophagy on oocytes or early embryos, leading to accumulation of damaged mitochondria. This is exacerbated by the increased activity of PIGBOS to form inter‐mitochondrial clusters, resulting in clumping. Such mitochondrial clumps may persist in early embryos making it difficult to be cleared off by mitophagy.

### 3.5 circRNAs in Regulating P‐Body Aging

Although circRNAs have been shown to accumulate in post‐mitotic cells, few studies have addressed their functional roles in regulating cellular or organismal aging (Kim et al. 2021; Weigelt et al. 2020; Westholm et al. 2014). Previous studies in *Drosophila* oocytes and brain have shown that P‐bodies condense during cellular aging and form viscous solid‐like condensates that promote translation repression (Pushpalatha et al. 2022; Sankaranarayanan et al. 2021). Our data is consistent with a model that *circdlg1* promotes P‐body coalescence during cellular aging. While it is unclear whether *circdlg1* regulates the biophysical states of P‐bodies, it is tempting to speculate that *circdlg1*‐mediated P‐body coalescence may regulate the translation of mRNAs important for proper cellular functions.

Our data suggest that during aging, *circdlg1* mis‐localizes and is recruited to P‐bodies, potentially leading to changes in P‐body dynamics and composition, which result in the displacement of *coch* mRNA from the P‐bodies. In future, more in‐depth genetic and biochemical studies are needed to verify this model. Recent studies have provided evidence that aging of condensates can lead to changes in their binding affinities to different mRNA (Kang et al. 2025; Milano et al. 2025). Further studies are needed to investigate the process of P‐body aging in granulosa cells using biophysical and biochemical methods.

Overall, our study identifies the spatiotemporal events during preovulatory follicle aging and how their coordinated actions lead to changes in oocyte quality in *Drosophila*. Mammalian and *Drosophila* oogenesis share many aspects that are highly conserved, thus our work may shed important insights on the general principles governing aging in preovulatory follicles (Deady et al. 2015; Greenblatt et al. 2019; Lei and Spradling 2016; Niu and Spradling 2022; Spradling et al. 2022). Guided by these novel findings in the orchestrated interactions between the oocyte and somatic cells, it is envisioned that stable and robust biomarkers like *circdlg1* are detected in human granulosa cells (somatic cells surrounding the human oocyte) and eventually blood, which can be accurately assessed to predict oocyte quality and aging which has direct impact on women's reproductive outcomes.

## 4 Materials and Methods

### 4.1 Fly Husbandry

The following fly strains were used in this study: *Oregon R* and *y w* (used as Control unless otherwise stated), *vasa‐Gal4* (Salzmann et al. 2013), *MTD‐Gal4* (Petrella et al. 2007), *GMR47A04‐Gal4* (Deady et al. 2015), *PIGBOS RNAi #1* and *#2* (this study), *PIGBOS Full Length OE* (this study), *PIGBOS C‐terminal OE* (this study), *PIGBOS N‐terminal OE* (this study), *circdlg1 RNAi #1 and #2* (this study), *sesn* RNAi *HMS05363* (BDSC:64027), *sesn‐myc* (Lu et al. 2021), *coch RNAi* (BDSC #35696, #41873, #60341), *coch‐GFP* (Vachias et al. 2025) and *EcR‐DN* (BDSC #6872). The flies were maintained in standard cornmeal medium at 25°C unless otherwise stated. Leucine was added to the fly food in as described previously (Gu et al. 2022).

### 4.2 Generation of Transgenic Flies

UASp‐PIGBOS CDS overexpression constructs were generated as described earlier using Gateway technology (Ng et al. 2024a; Wong et al. 2017). Transgenic flies were generated by BestGene using P‐element mediated insertion. Generation of shRNAs against the unique exon of PIGBOS and back‐spliced junction of *circdlg1* was performed as described previously (Ng et al. 2024a; Ng and Pek 2021; Pek et al. 2015). shRNAs were cloned into Valium22 (for PIGBOS) and Valium20 (for *cirdlg1*) and inserted into flies using the phiC31 integrase‐mediated integration by BestGene.

### 4.3 Oocyte Aging Assay

Aging assay was performed as previously described with some modifications (Greenblatt et al. 2019; Greenblatt and Spradling 2018). Female virgin flies were placed in standard cornmeal medium added with yeast paste made by mixing yeast powder with sterile water until the mixture reached a peanut butter consistency. After feeding for 24–64 h, virgin flies were placed in apple juice vials (3% agarose, 25% apple juice, 0.3% sucrose, 0.03% Tegosept, water) and aged for 2–12 days. The virgins were transferred to new apple juice vials every 2 to 3 days. To study the oocyte quality, aged virgin flies were transferred to standard cornmeal medium containing control male flies in equal numbers. Males were isolated 2 days prior to mating, and only 4 to 8 days old males were used. Laid eggs were counted after 16 or 24 h of mating followed by counting the unhatched eggs 24 h later to determine the hatch rates as described previously (Ng et al. 2024b; Voo et al. 2021).

### 4.4 Defolliculation of Stage 14 Oocytes

Defolliculation was performed as described previously (Greenblatt et al. 2019; Sieber et al. 2016). Ovaries were dissected in Grace Insect's medium (Gibco). The medium was replaced with 10% bleach (Clorox) and mixed gently for 10–20 s. The bleach solution was diluted using sterile water that was twice its volume and centrifuged at 1000 x g, 4°C for 1 min. The defolliculated oocytes were then filtered using a 70 μm cell strainer (VWR) and rinsed with cold 1X PBS. Under the microscope, oocytes that sank were collected and used for the downstream experiments.

### 4.5 Mitochondria Fractionation

Fractionation was performed using the Mitochondria Isolation Kit for Cultured Cells (Thermo Scientific) with some modifications to the manufacturer's protocol. Sixty pairs of ovaries were dissected and defolliculated. The defolliculated oocytes were incubated in Mitochondria Isolation Reagent A containing 1X Halt protease inhibitor cocktail (Thermo Scientific) for a maximum of 2 min and homogenized using a motorized pellet homogenizer. Then, an equal volume of Mitochondria Isolation Reagent C supplemented with 1X Halt protease inhibitor cocktail (Thermo Scientific) was added to the lysed oocytes and centrifuged at 700 *x* g for 10 min at 4°C. The supernatant was carefully transferred to a new tube and centrifuged at 3000 *x* g, 4°C for 15 min to obtain more purified mitochondria. The resulting supernatant was collected and labeled as the cytosol fraction and the pellet as the mitochondria fraction. The pellet was washed twice using Mitochondria Isolation Reagent C containing 1X Halt protease inhibitor cocktail (Thermo Scientific) and centrifuged at 12,000 *x* g, 4°C for 5 min. The pellet was resuspended with Protein Extraction Buffer (50 mM Tris–HCl pH 7.5, 150 mM NaCl, 5 mM MgCl_2_, 0.1% NP‐40) supplemented with Protease Inhibitor Cocktail (Roche). The samples were kept at −80°C for downstream experiments.

### 4.6 Immunostaining

Immunostaining was carried out as described previously (Osman and Pek 2018; Wong et al. 2017). Briefly, ovaries were fixed in Grace Insect's medium (Gibco) containing 4% PFA (Electron Microscopy Sciences) for 10–20 min. The ovaries were washed three times with 1X PBS‐T (0.2% Triton X‐100) for 10 min followed by blocking with 5% normal goat serum for a minimum of 30 min. Ovaries were incubated with primary antibody mix overnight at room temperature with shaking (350 rpm). Primary antibodies used were as follows: mouse anti‐ATP5a (1:300, Abcam 15H4C4), rabbit anti‐FLAG (1:500, Sigma‐Aldrich F7425), rabbit anti‐p62 (1:1000, gift from Shoichiro Kurata) and Tamaki Yano (Nagai et al. 2021), mouse anti‐Arm (1:100, DSHB N2 7A1), rabbit anti‐aPKC (1:1000, Santa Cruz SC‐208), mouse anti‐Me31B (1:2000, gift from Aikra Nakamura), rat anti‐TraI (1:500, gift from Elmar Wahle (Gotze et al. 2017)) and mouse anti‐GFP (1:1000, Invitrogen 3E6 A‐11120). The next day, the ovaries were washed three times with 1X PBS‐T and incubated with secondary antibody mix for 4 h at room temperature with shaking (350 rpm). DNA was stained with DAPI, and ovaries were mounted in Vectashield Anti‐fade Mounting Medium (Vector Laboratories) and examined under Upright Olympus FV3000.

### 4.7 Single Molecule Fluorescent In Situ Hybridization (smFISH)

smFISH for *circdlg1* was performed using the BaseScope Assay v2 RED kit (ACDbio, 323,910) as previously described (Ng et al. 2024a). Probes were designed to specifically target the *circdlg1* back‐spliced junction. For granulosa cells, a different fixation protocol was performed. Samples were fixed in 1% paraformaldehyde at 4°C over 48–60 h. The samples were then washed with 0.2% PBX‐Triton and subjected to dehydration and rehydration. They were incubated in a series of 50%, 75%, 100%, 75%, 50% and 25% methanol in PBS for 5 min each, and rinsed in PBX before proceeding with the BaseScope protocol. The samples were mounted in Vectashield and examined under the Olympus FV3000 Upright confocal microscope.

### 4.8 S2 Cells Transfection

Serum‐independent Schneider 2 (S2) cells (Steve Cohen's laboratory) were cultured at 25°C in Express Five serum‐free medium (SFM) (Gibco) and sub‐cultured once every week. For transfection, S2 cells with > 90% confluency were transfected with 1.5 μg of plasmid DNA together using Cellfectin II reagent (Gibco) in accordance with the manufacturer's protocol in a 6‐well plate as previously described (Chan and Pek 2022). The cells were harvested after 48 h of incubation at 25°C. Harvested cells were washed with cold 1X PBS and lysed in Protein Extraction Buffer using a motorized pellet homogenizer. The cell lysate was kept at −80°C and used for downstream experiments.

### 4.9 Immunoprecipitation

Immunoprecipitation was done as described previously (Chan and Pek 2022). S2 cells collected were homogenized and lysed in Protein Extraction Buffer (50 mM Tris–HCl pH 7.5, 150 mM NaCl, 5 mM MgCl_2_, 0.1% NP‐40) supplemented with Protease Inhibitor Cocktail (Roche). The lysate was pre‐cleared by blocking with protein A/G agarose beads (Merck Millipore). Antibodies were added and incubated overnight at 4°C with agitation. For negative control, no antibody or IgG was added instead. On the next day, fresh protein A/G beads were added and incubated for two hours at 4°C followed by washing three times using Protein Extraction Buffer. Thereafter, protein or RNA was extracted using 2X Sample Buffer (Bio‐Rad) containing beta‐mercaptoethanol (Sigma‐Aldrich) or Direct‐zol RNA miniprep kit (Zymo Research) respectively.

### 4.10 Generation of PIGBOS Antibody

The generation of PIGBOS antibody was outsourced to GenScript Biotech Corporation. The antibody was generated against *Drosophila melanogaster* PIGBOS‐PE using the following peptide sequence: KYKNEKKTEAETPAC (Flybase ID: FBpp0312560; UniProt: A0A0S0WGS4) in rabbits. The specificity of the antibody was checked for subsequent application in western blotting (Figure S1B).

### 4.11 Western Blotting

Western blotting was performed as described previously with modifications (Tay and Pek 2017). Ovaries and S2 cells were homogenized and lysed in Protein Extraction Buffer (50 mM Tris–HCl pH 7.5, 150 mM NaCl, 5 mM MgCl_2_, 0.1% NP‐40) supplemented with Protease Inhibitor Cocktail (Roche). 2X Sample Buffer (Bio‐Rad) containing beta‐mercaptoethanol (Sigma‐Aldrich) was added to the desired volume for loading in a 12% polyacrylamide gel. Primary antibodies used were rabbit anti‐PIGBOS (1:500, this study), mouse anti‐ATP5A (1:1000, Abcam 15H4C4), mouse anti‐Actin (1:200, DSHB JLA20), mouse anti‐FLAG (1:2000, Sigma‐Aldrich F7425), anti‐myc (1:1000, Sigma‐Aldrich clone 4A6), rabbit anti‐pS6K (1:1000, Cell Signaling #9209) and guinea pig anti‐S6K (1:3000, gift from Aurelio Teleman) (Hahn et al. 2010). Actin or total protein was used as the loading control. The blots were detected using the ChemiDoc Touch Imaging System (BioRad) under non‐saturating conditions. The band intensities were quantified using ImageJ software and normalized to Actin or total protein.

### 4.12 RNA Extraction

*Drosophila* ovaries were dissected in cold Phosphate Buffered Saline (PBS) and transferred to 1.5 mL Eppendorf tubes. The collected ovaries or oocytes were homogenized in TRIzol (Invitrogen) using a motorized pestle homogenizer and RNA was extracted using the Direct‐zol miniprep kit (Zymo Research) as described previously (Pek et al. 2015). The RNA was quantified using a Nanodrop spectrophotometer (Thermo Scientific).

### 4.13 RT‐PCR and RT‐qPCR

RT‐PCR and qRT‐PCR were done as previously described (Pek et al. 2015; Tay and Pek 2019). RT was performed using random hexamers as primers with the M‐MLV Reverse Transcriptase (Promega). PCR was carried out to determine the RT efficiency using *actin5C* primers. PCR products were run on a 1% agarose gel for visualization. *circdlg1* was detected using outward‐pointing primers previously reported (Westholm et al. 2014). *Actin5C* was used as a loading control. KAPA SYBR Fast qPCR Master Mix kit (2X) (KAPA Biosystems) was used on the QuantStudio 5 System (Life Technologies). Oligonucleotides used are available in Table 1.

**TABLE 1:** Primer sequences.

Primer name	5′‐3′ sequence
*Actin5C* Fw	TGCCCATCTACGAGGGTTAT
*Actin5C* Rv	AGTACTTGCGCTCTGGCGG
*PIGBOS* Fw	GCCATGCAAATTCGTCGT
*PIGBOS* Rv	TCGTTGTGGCGAATCTTT
*cup* Fw	CAACGCTGGAGCTGTCAATA
*cup* Rv	GAAGAGGTACTCCGCACTGG
*ND4* Fw	CAGAACGTTTACAAGCTGGTT
*ND4* Rv	CAGAAACTGGAGCTTCAACATG
*ND5* Fw	TCGAATTGGGGATGTAGCTTT
*ND5* Rv	AGCAGAAACAGGTGTAGGAGC
*Cyt‐b* Fw	AGTAACACCTGCCCATATTCAA
*Cyt‐b Rv*	GGTCGAGCTCCAATTCAAGTTAA
*CoI* Fw	TGACTTCTACCTCCTGCTCTTTC
*CoI* Rv	GCAATTCCAGCGGATAGAGGT
*CoII* Fw	TGATAACCGAGTAGTTTTACCCA
*CoII* Rv	ACCATAAAATAAACCCGGTCGA
*CoIII* Fw	CACGAGAAGGAACATACCAAGGA
*CoIII* Rv	AGCGGGTGATAAACTTCTGTGA
*circdlg1* Fw	ACAATTGTCGCAGTCCCAAT
*circdlg1* Rv	ACCAGCTATCATCGCCATTC
*Dlg1* Fw	CCAAGTTGCCCAGCTGTATC
*Dlg1* Rv	CCGCTCGTAAGTCTTCTTGG
*circmbl1* Fw	AGGACACCGAATGCAAGTTC
*circmbl1* Rv	AAACGCAGCTGTTAATTTTTG
*Coch mRNA Fw1*	TTCACCCTGCAGTTCTACGT
*Coch mRNA Rv1*	AGCGAGATGAAAGGTCCCAA

### 4.14 RNA Sequencing and Analysis

Library construction, sequencing and analysis were outsourced to NovogeneAIT Genomics. For linear RNA sequencing, rRNA‐depletion was performed using the Ribo‐Zero kit, followed by library construction and sequencing (Illumina). After quality control, the reads were mapped using HISAT2 software (Kim et al. 2015). The mapped reads were assembled by StringTie and ballgrown was used to detect differential expressed transcripts (*p* < 0.05) (Pertea et al. 2016). For circRNA sequencing, additional RNase R treatment to digest linear RNA was performed. The sequencing libraries were generated by NEBNext UltraTM Directional RNA Library Prep Kit for Illumina following the manufacturer's protocol. Reads were mapped using the Spliced Transcripts Alignment to a Reference (STAR) software, and circRNAs identified using CIRCexplorer2 (Dong et al. 2019). Differential expression analysis was done using DESeq2.

### 4.15 *In silico* Prediction of the Transmembrane Domains

PIGBOS transmembrane domains were predicted by inputting PIGBOS FL amino acid sequence (MQIRRPRGVT VPQLMVVTAI GLLGGIYIWQ PLILKYKNEK KTEAETPAVT ETSGTTNFIN GFKRFATTTV GLMAIGIGST VIFYTTHRLV IKPYLLEKRR LEAEASAEYL FQQEVHSQIG ESRPKRSEY) into the TOPCONS web server (Tsirigos et al. 2015). The predicted protein structure was illustrated using SWISS‐MODEL Workspace (Waterhouse et al. 2018).

### 4.16 Quantification and Statistical Analyzes

Results were analyzed statistically using GraphPad Prism software (GraphPad Software Inc). Student *T*‐tests were performed for results containing *p*‐values, and all error bars were indicated using standard deviation (SD) values. The number of independent biological replicates was indicated in the figure legends. Quantification of the band intensity in either protein blots or DNA gels was performed using ImageJ. Quantification of P‐body number and size was done using Imaris software.

## Author Contributions

Performed experiments, data acquisition: X.Y.K., K.J.T., Z.H.L., S.C.O., S.E.T., J.X.L., J.W.P. Data analysis and investigation: X.Y.K., K.J.T., J.W.P. Methodology: X.Y.K., K.J.T., Z.H.L., J.W.P. Writing – original draft: X.Y.K., J.W.P. Writing – review and editing: X.Y.K., K.J.T., Z.H.L., S.C.O., S.E.T., J.X.L., Z.H., J.W.P. Conceptualization: J.W.P. Supervision: J.W.P. Funding acquisition: Z.H., J.W.P. Project administration: J.W.P.

## Funding

This work was supported by Temasek Life Sciences Laboratory (Grant 3170) and Seah Cheng Siang Memorial Research Award 2021.

## Conflicts of Interest

The authors declare no conflicts of interest.

## Supporting information


**Figure S1:** Somatic knockdown of Ecdysone signaling pathway promotes preovulatory follicle aging.

**Figure S2:** Analyzes of PIGBOS antibody and *PIGBOS* RNAi.

**Figure S3:** Overexpression of PIGBOS rescues *PIGBOS* RNAi aging phenotype.

**Figure S4:** Analyzes of PIGBOS protein *in silico* and interaction.

**Figure S5:** Analyzes of *circdlg1*.

## Data Availability

The data that support the findings of this study are openly available in Gene Expression Omnibus (GEO), reference number GSE283504.
