# Supplementary material for: Spatiotemporal Crosstalk Between Oocyte and the Microenvironment Governs Preovulatory Follicle Aging
Source: Aging Cell. 2025 Nov 23;25(1):e70302. doi: 10.1111/acel.70302 (PMC12740097; doi:10.1111/acel.70302)
Supplement: Supplementary file 1 — Figure S1: Somatic knockdown of Ecdysone signaling pathway promotes preovulatory follicle aging. [file ACEL-25-e70302-s003.docx]

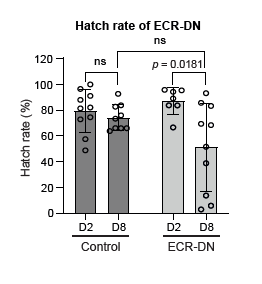


**Figure S1. Somatic knockdown of Ecdysone signaling pathway promotes preovulatory follicle aging.**

*GMR47A04>Ecdysone Receptor-Dominant Negative* (*ECR-DN*) flies show reduced hatch rate at Day 8 (D8) when compared to Day 2 (D2) but not in control (*y w*) flies. Data are mean values +/- SD. *P* values were calculated using two tailed Student’s t-test with ns = not significant.
