# Supplementary material for: Spatiotemporal Crosstalk Between Oocyte and the Microenvironment Governs Preovulatory Follicle Aging
Source: Aging Cell. 2025 Nov 23;25(1):e70302. doi: 10.1111/acel.70302 (PMC12740097; doi:10.1111/acel.70302)
Supplement: Supplementary file 2 — Figure S2: Analyzes of PIGBOS antibody and PIGBOS RNAi. [file ACEL-25-e70302-s001.docx]

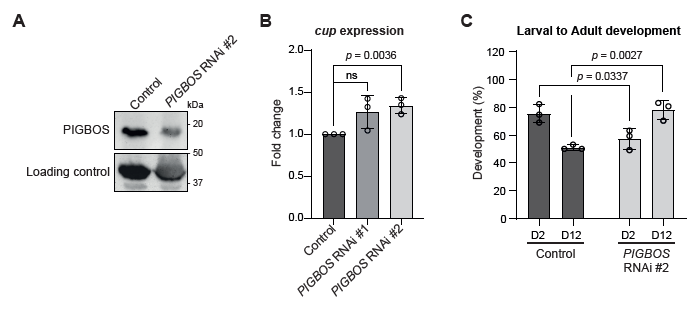


**Figure S2. Analyses of PIGBOS antibody and *PIGBOS* RNAi.**

(A) PIGBOS protein expression is downregulated in *PIGBOS* RNAi ovaries as compared to control (*Oregon R*), showing specificity of antibody.

(B) *PIGBOS* RNAi did not knockdown *cup* mRNA. Data are presented as mean values +/- SD with n = 3 biological replicates. The exact *p*-value shown was obtained using Student’s T-test with ns = not significant.

(C) Improved in larval to adult development of *PIGBOS* RNAi #2 as compared to control (sibling control) after 12 days (D12) of aging. Data are presented as mean values +/- SD with n = 3 biological replicates. The exact *p*-value shown was obtained using Student’s T-test with ns = not significant.
