# Supplementary material for: Spatiotemporal Crosstalk Between Oocyte and the Microenvironment Governs Preovulatory Follicle Aging
Source: Aging Cell. 2025 Nov 23;25(1):e70302. doi: 10.1111/acel.70302 (PMC12740097; doi:10.1111/acel.70302)
Supplement: Supplementary file 3 — Figure S3: Overexpression of PIGBOS rescues PIGBOS RNAi aging phenotype. [file ACEL-25-e70302-s005.docx]

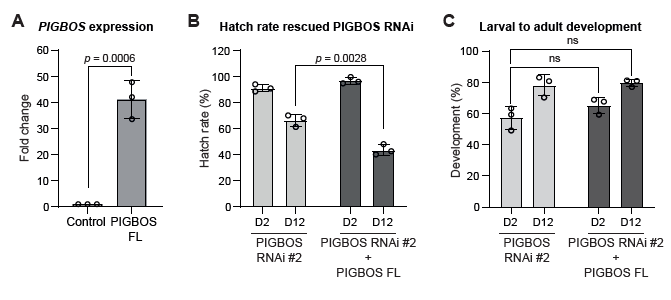


**Figure S3. Overexpression of PIGBOS rescues *PIGBOS* RNAi aging phenotype.**

(A) The relative expression of *PIGBOS* in PIGBOS FL overexpression compared to control (*Oregon R*). Data are presented as mean values +/- SD with n = 3 biological replicates. The exact *p*-value shown was obtained using Student’s T-test.

(B) Hatch rate decreases when *PIGBOS* RNAi flies are rescued with PIGBOS FL after 12 days (D12) of aging but not after 2 days (D2). Data are presented as mean values +/- SD with n = 3 biological replicates. The exact *p*-value shown was obtained using Student’s T-test.

(C) The larval to adult development shows no significant differences in *PIGBOS* RNAi with or without PIGBOS FL. Data are presented as mean values +/- SD with n = 3 biological replicates. The exact *p*-value shown was obtained using Student’s T-test with ns = not significant.
