# Supplementary material for: Spatiotemporal Crosstalk Between Oocyte and the Microenvironment Governs Preovulatory Follicle Aging
Source: Aging Cell. 2025 Nov 23;25(1):e70302. doi: 10.1111/acel.70302 (PMC12740097; doi:10.1111/acel.70302)
Supplement: Supplementary file 4 — Figure S4: Analyzes of PIGBOS protein in silico and interaction. [file ACEL-25-e70302-s002.docx]

**
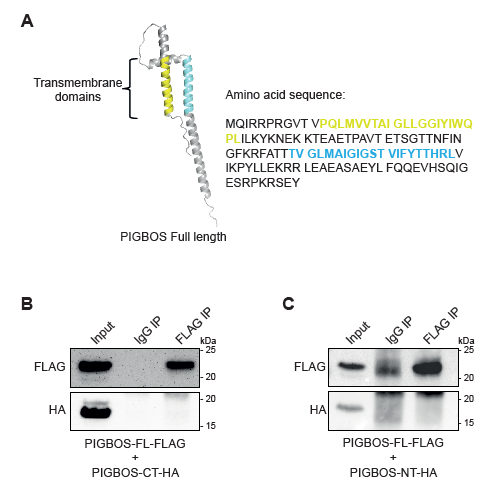
**

**Figure S4. Analyses of PIGBOS protein *in silico* and interaction.**

(A) *In silico* analysis of PIGBOS showing the transmembrane domains (highlighted in yellow and cyan) predicted using TOPCONS web server.

(B,C) Co-immunoprecipitation showing that PIGBOS-FL does not bind to PIGBOS-CT and NT.
