# Supplementary material for: Spatiotemporal Crosstalk Between Oocyte and the Microenvironment Governs Preovulatory Follicle Aging
Source: Aging Cell. 2025 Nov 23;25(1):e70302. doi: 10.1111/acel.70302 (PMC12740097; doi:10.1111/acel.70302)
Supplement: Supplementary file 5 — Figure S5: Analyzes of circdlg1. [file ACEL-25-e70302-s004.docx]

**
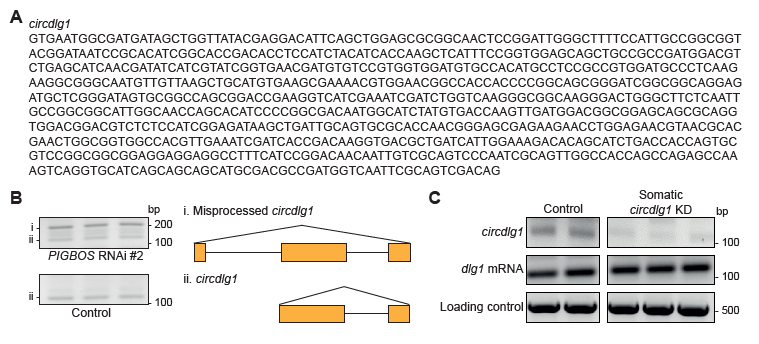
**

**Figure S5. Analyses of *circdlg1*.**

(A) Full-length sequence of *circdlg1*.

(B) Misprocessed *circdlg1* (band i) is detected in *PIGBOS* RNAi flies but absent in control (*Oregon R*) flies. Sequencing of band i shows that a different form of *circdlg1* is produced by back-splicing at a different exon. Both genotypes show the presence of *circdlg1* (band ii).

(C) RT-PCR showing specific knockdown of *circdlg1* in ovaries of *GMR47A04>circdlg1* RNAi #2.
